# Cannabidiol and Other Non-Psychoactive Cannabinoids for Prevention and Treatment of Gastrointestinal Disorders: Useful Nutraceuticals?

**DOI:** 10.3390/ijms21093067

**Published:** 2020-04-26

**Authors:** Vicente Martínez, Amaia Iriondo De-Hond, Francesca Borrelli, Raffaele Capasso, María Dolores del Castillo, Raquel Abalo

**Affiliations:** 1 Department of Cell Biology, Physiology and Immunology, Neurosciences Institute, Universitat Autònoma de Barcelona, 08193 Bellaterra, Barcelona, Spain; vicente.martinez@uab.es; 2 Centro de Investigación Biomédica en Red de Enfermedades Hepáticas y Digestivas (CIBERehd), Instituto de Salud Carlos III, 28049 Madrid, Spain; 3 Instituto de Investigación en Ciencias de la Alimentación (CIAL) (UAM-CSIC), C/Nicolás Cabrera, 9, Campus de la Universidad Autónoma de Madrid, 28049 Madrid, Spain; amaia.iriondo@csic.es (A.I.D.-H.); mdolores.delcastillo@csic.es (M.D.d.C.); 4 Department of Pharmacy, School of Medicine and Surgery, University of Naples Federico II, Via Domenico Montesano 49, 80131 Naples, Italy; franborr@unina.it; 5 Department of Agricultural Sciences, University of Naples Federico II, Via Università 100, 80055 Portici (NA), Italy; 6 High Performance Research Group in Physiopathology and Pharmacology of the Digestive System NeuGut-URJC, Department of Basic Health Sciences, Faculty of Health Sciences, Universidad Rey Juan Carlos (URJC), Campus de Alcorcón, Avda. de Atenas s/n, 28022 Madrid, Spain; 7 Unidad Asociada I+D+i del Instituto de Química Médica (IQM), Consejo Superior de Investigaciones Científicas (CSIC), Madrid, Spain; raquel.abalo@urjc.es

**Keywords:** cannabidiol, cannabinoids, inflammatory bowel disease, irritable bowel syndrome, gastrointestinal, non-psychoactive cannabinoids, nutraceutical, psychoactive cannabinoids, visceral pain

## Abstract

*Cannabis sativa* is an aromatic annual flowering plant with several botanical varieties, used for different purposes, like the production of fibers, the production of oil from the seeds, and especially for recreational or medical purposes. Phytocannabinoids (terpenophenolic compounds derived from the plant), include the well-known psychoactive cannabinoid Δ^9^-tetrahydrocannabinol, and many non-psychoactive cannabinoids, like cannabidiol. The endocannabinoid system (ECS) comprises of endocannabinoid ligands, enzymes for synthesis and degradation of such ligands, and receptors. This system is widely distributed in the gastrointestinal tract, where phytocannabinoids exert potent effects, particularly under pathological (i.e., inflammatory) conditions. Herein, we will first look at the hemp plant as a possible source of new functional food ingredients and nutraceuticals that might be eventually useful to treat or even prevent gastrointestinal conditions. Subsequently, we will briefly describe the ECS and the general pharmacology of phytocannabinoids. Finally, we will revise the available data showing that non-psychoactive phytocannabinoids, particularly cannabidiol, may be useful to treat different disorders and diseases of the gastrointestinal tract. With the increasing interest in the development of functional foods for a healthy life, the non-psychoactive phytocannabinoids are hoped to find a place as nutraceuticals and food ingredients also for a healthy gastrointestinal tract function.

## 1. Introduction

Dietary effects of nutraceuticals on gastrointestinal (GI) health are well recognized, and specific diets have been related to the prevention or the reduction of incidence of certain GI pathologies. Moreover, specific food ingredients, mediating these effects, have been characterized and isolated, and their efficacy assessed experimentally. This evidence supports the general use of nutraceuticals and food ingredients for the treatment and prevention of GI diseases or the overall promotion of GI health [1]. Moreover, the wide acceptance and use of complementary and alternative medicine by patients with inflammatory and functional GI disorders [2,3] further supports the interest in developing nutraceuticals targeting the GI tract.

**Figure 1:**
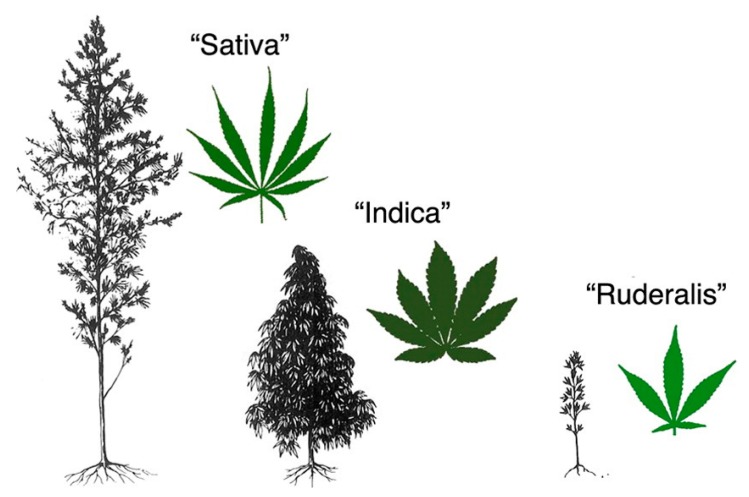
Morphological differences among varieties of *Cannabis sativa* species, image from John M. McPartland. Cannabis and Cannabinoid Research. Dec 2018.203-212. http://doi.org/10.1089/can.2018.0039.

Among the possible plant-derived nutraceuticals for treatment of GI disorders are those from Cannabis. Cannabis is a generic term used to indicate preparations obtained from the plant *Cannabis sativa*, an aromatic annual flowering herb. There is debate and confusion over the taxonomic organization of *Cannabis.* However, John M. McPartland concluded in a scientific review that the family *Cannabaceae* includes genera *Cannabis*, *Humulus*, and *Celtis*. In the genus *Cannabis*, the species *sativa* has three varieties, *sativa*, *indica* and *ruderalis* [4], which are discussed in the present review. Figure 1 shows de morphologic differences between the three varieties of *Cannabis sativa*. Importantly, depending on the variety, *Cannabis sativa* can be used for several purposes, including the production of fibers, the production of oil from the seeds, and mainly for recreational or medical purposes.

This plant contains over 500 chemical compounds, and more than 120 of them are terpenophenolics, also named phytocannabinoids [5,6]. The most studied, and therefore, the most known phytocannabinoids, are the psychoactive cannabinoid Δ^9^-tetrahydrocannabinol (THC) and the non-psychoactive cannabinoids: cannabidiol (CBD), cannabigerol (CBG), cannabichromene (CBC) and cannabidivarin (CBDV). 

In the last forty years, phytocannabinoids have attracted considerable attention for their biological activity beneficial to human health, such as appetite-stimulant, antiemetic, anti-spasticity, analgesic, anti-inflammatory, and antitumoral properties. The mechanism responsible for the phytocannabinoids effects has been unknown until the discovery of the endocannabinoid system (ECS), in the early 1990s. The GI tract contains all the elements of the ECS (endocannabinoid ligands, synthesis and degradation enzymes of such ligands, and receptors), and thus, phytocannabinoids may powerfully impact on this system. With the increasing interest in the development of functional foods for a healthy life, there is hope that the non-psychoactive phytocannabinoids will find a place as nutraceuticals and food ingredients also for a healthy GI tract function.

In this narrative review, we will first look at the hemp plant as a possible source of new functional food ingredients and nutraceuticals that might be eventually useful to treat or even prevent GI conditions. Then, we will briefly describe the ECS and will summarize the general pharmacology of phytocannabinoids, which are present at different proportions in the different *Cannabis sativa* varieties. Finally, we will revise the available data showing that non-psychoactive phytocannabinoids, particularly CBD, may be useful to treat different disorders and diseases of the GI tract.

## 2. Use of Hemp and Non-Psychoactive Phytocannabinoids as Nutraceuticals and Food Ingredients

The *Cannabis sativa* edibles industry will be a combination of the food and the pharmaceutical industries. According to the Agricultural Marketing Act of 1946, hemp is defined as “the plant *Cannabis sativa* and any part of that plant, including the seeds thereof and all derivatives, extracts, cannabinoids, isomers, acids, salts, and salts of isomers, whether growing or not, with a THC concentration of not more than 0.3 percent on a dry weight basis” [7]. The cultivation and consumption of hemp seeds with low (<0.3%) THC levels has been recently legalized in Australia, Canada and the United States, and there is a growing interest in hemp seed, due to its nutritional value [8]. Breeding of different *Cannabis sativa* varieties for low THC levels has been a main target in hemp breeding, and levels below 0.2% THC have been reached for some cultivars. Regulations that allowed a THC content of only 0.2% were implemented in the European Union in 2001. Since then, a further and stable reduction of THC has gained importance as a breeding goal [9]. However, further research is required to ensure the quality, safety and beneficial properties of hemp food products.

The taxonomic organization of the genus *Cannabis* has been described above (see Section 1). Here we will concentrate on describing the features that make this plant attractive for the development of new nutraceuticals and food ingredients that might be useful for a healthy life, in general, and a healthy GI tract function, in particular.

**Figure 2:**
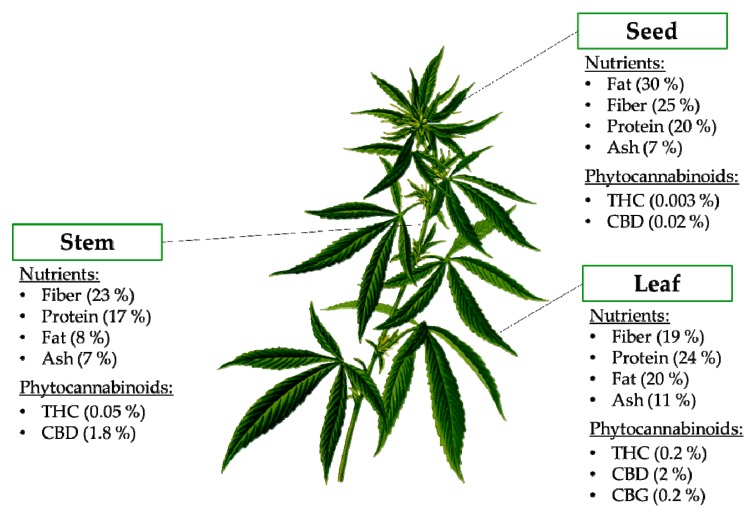
Nutritional composition and phytocannabinoids present in the different anatomic parts of the hemp plant. Abbreviations: CBD, cannabidiol; CBG, cannabigerol; THC, Δ^9^-tetrahydrocannabinol.

Figure 2 shows the different anatomic parts of the hemp plant and the presence of nutrients and bioactive compounds in each part. Hemp seeds have been studied extensively in the past, but little is known on the composition and functional characteristics of the other anatomic parts of the hemp plant. More recently, the nutritional composition of the leaves and stem of Cannabis sativa have been described [10]. The major component present in the stem was fiber (23.14%) followed by protein, fat and ash (Figure 2) [11]. The leaf showed a lipid content of 19.97%. The crude protein content in the leaf was 23.78%, while the crude fiber was 18.95%, and the ash content was 11.18% [11]. These authors also reported the amino acid profile of the different parts of the plant and nine of the ten essential amino acids (lysine, histidine, arginine, threonine, valine, methionine, isoleucine, leucine and phenylalanine) were found in the leaves of *C. sativa*. The amino acid profile of *C. sativa* leaves is comparable in amino acid content to that from hemp seed and even egg white and soybeans [11].

The most abundant bioactive compounds found in hemp are Δ^9^-tetrahydrocannabinolic acid (THCA), cannabidiolic acid (CBDA) and cannabinolic acid (CBNA), followed by cannabigerolic acid (CBGA), cannabichromenic acid (CBCA) and cannabinodiolic acid (CBNDA) [12]. Phytocannabinoids accumulate in female flowers and in most aerial parts, but they have also been detected in low quantity in other parts of the plant (Figure 2). Generally, the concentration of these compounds depends on tissue type, age, variety, growth conditions (nutrition, humidity, light level), harvest time and storage conditions [12]. Cannabinoids in leaves have been shown to decrease with age, and along the stem axis, with the highest levels observed in the leaves of the uppermost nodes [13].

Since the consumption of hemp seeds has been legalized in some countries, their nutritional composition has been deeply studied. Hemp protein is mainly composed of globulin and albumin, and is characterized for its exceptionally high level of arginine and glutamic acid [8]. According to the FoodData Central database of the United States Department of Agriculture (USDA), hemp seed is composed of approximately 30–50% oil, of which 80% corresponds to unsaturated fatty acids. Hemp seed oil is composed of essential fatty acids, such as linoleic acid, alpha-linolenic acid and oleic acid [8]. Compared to other plant oils, hemp seed oil has the highest proportion of polyunsaturated fatty acids, which have the potential to reduce the risk of cardiovascular diseases, cancer, rheumatoid arthritis, hypertension, inflammatory and autoimmune diseases [8,14,15]. The omega-6 to omega-3 ratio (n6/n3) in hemp seed oil is normally between 2:1 and 3:1, which is considered to be optimal for human health [14]. In addition, hemp seed also contains both soluble and insoluble dietary fiber at a ratio about 20:80 [14]. Specifically, the insoluble dietary fiber of hemp seed is composed of cellulose (46%), lignin (31%) and hemicellulose (22%) [15].

The whole hemp plant is a source of fibers, oil and bioactive compounds, and therefore, it is a good candidate to be used for human nutrition. The main limitation of using hemp for human consumption is the content of the psychoactive cannabinoid THC. Its precursor THCA is thermally unstable and can be decarboxylated when exposed to light or heat [16]. The transformation of THCA into THC could occur during food storage and processing affecting food safety. Therefore, the selection of THC-free and low THCA species is of great interest to ensure the safety of hemp-based products. However, in many cases, the amounts of THC detected in products that are marketed today are greater than expected when using certified species with levels of this compound within the established (<0.2%) for its production for food consumption. Specific advanced analytical methods should be implemented to avoid the decarboxylation of THCA when quantifying THC. There is much criticism around the use of gas chromatography for cannabinoid analysis, since the high temperature of both injector and detector lead to decarboxylation of cannabinoid acids if not previously derivatized [17].

Hemp and its derivatives can be used as nutraceuticals or as food ingredients enriched in nutrients and non-nutrient bioactive compounds. Since *Cannabis* has a particular composition in nutrients and bioactive compounds, the exploitation of this plant in human nutrition is a priority research line at a global level. In 1989, Dr. Stephen DeFelice defined the term nutraceutical as “a substance that is a food or part of a food that provides medical and/or health benefits, including the prevention and treatment of disease” generally sold in medicinal forms (pills or tablets) not usually associated with foods [18]. This term is often interchanged with “functional food” in the scientific literature [19]. The European Commission Concerted Action on Functional Food Science in Europe (FUFOSE) defines functional foods as “a food, which beneficially affects one or more target functions in the body, beyond adequate nutritional effects, in a way that is relevant to either an improved state of health and well-being and/or reduction of risk of disease”. A functional food can be a natural food or a food to which a component has been added or removed by technological or biotechnological means, and it must demonstrate their effects in amounts that can normally be expected to be consumed in the diet [20].

Hemp and hemp-derived ingredients can be used as a source of nutrients and bioactive compounds with potential health claims. Due to the legalization to cultivate low-THC hemp, an increasing number of hemp seed food products have been developed by researchers and manufacturers. There is a wide variety of food products in which cannabinoid extracts have been added, such as beverages (dairy and non-dairy), breakfast cereals, cookies, brownies, vegan burgers and sausages, and even more recently, beer, wine, hemp-infused milks, barley-based sodas, health beneficial-honeys, and fortified sports products (Table 1) [21]. To date, 680 branded food products containing hemp seed derivatives in the form of oil, extract, flour or powder, are registered in the FoodData Central database of the USDA [22]. Any of these products consumed in the USA must not contain more than 0.3% THC to be legal by US government regulations [21].

In Europe, CBD food products that were legally on the market prior to January 2018 can continue to be sold as long as an application for novel food approved status is submitted. In the European Union (EU), the cultivation of *Cannabis sativa* varieties is permitted provided they are registered in the EU’s ‘Common Catalogue of Varieties of Agricultural Plant Species’, and the THC content does not exceed 0.2%. Some products derived from the *Cannabis sativa* plant or plant parts, such as seeds, seed oil, hemp seed flour, and defatted hemp seed, have a history of consumption in the EU [23]. However, any other product derived from hemp different from the seeds is considered novel and needs to undergo European Food Safety Authority’s (EFSA) authorization procedure according to the Regulation (EU) 2015/2283 before it could be introduced into the European food market. A novel food is defined as “food that was not used for human consumption to a significant degree within the Union before 15 May 1997, irrespective of the dates of accession of the Member States to the Union” [24]. Currently, only one application can be found on the European Commission Summary of the ongoing applications web page. The application for the authorization of trans-CBD contains safety and toxicological information and toxicity reviews, which include acute and long term toxicity studies in animals, and tolerance studies in humans [25].

In food, there are many potential applications for the use of hemp-based ingredients that have been discussed in the previous paragraphs. From a legal perspective, the future is still uncertain, and the regulation of hemp consumption is expected to be “a headache” during 2020 and the following years. New varieties with less THC and THCA content and non-thermal food processing methods are needed for the use of hemp as a novel food ingredient. There is the need to obtain the necessary and sufficient scientific evidence to certify the safety and to assign nutrition and health claims of food containing hemp-based ingredients. Further research is required to ensure the quality, safety and beneficial properties of hemp food products. Finally, more research regarding the health-promoting properties of pure and combined bioactive compounds in hemp is also needed.

## 3. The Endogenous Cannabinoid System

The ECS is a cell-signaling system, comprising endogenous ligands (endocannabinoids), cannabinoid receptors (CB1 and CB2) and proteins involved in endocannabinoid synthesis and inactivation [26,27].

Cannabinoid receptors (CB1 and CB2) are G protein-coupled receptors (GPR) containing seven transmembrane spanning domains. The CB1 receptor, identified in the brain in 1988 [28], is predominantly localized in the neurons of the central nervous system (CNS) on presynaptic terminals, where it mediates retrograde signaling of endocannabinoids. To a lesser extent, CB1 is also localized in microglia, astrocytes, and oligodendrocytes of the CNS [29,30]. In addition to CNS, CB1 is also expressed in the peripheral nervous system (PNS) and peripheral tissues. In the PNS, CB1 is localized in sympathetic nerve terminals, and more importantly, in primary sensory neurons where it affects nociception from afferent nerve fibers [31]. In the GI tract, CB1 is expressed in the enteric nervous system (ENS) and in non-neural cells where it regulates the GI functions (i.e., motility, secretion and permeability) and energy balance [32]. In the cardiovascular system, CB1 is expressed in specific restricted regions within cardiac myocytes, where it seems to be involved in cardiovascular dysfunctions [33,34]. The CB1 receptor is also localized in the liver, lung, bone, skin, eye, adipose tissue and skeletal muscle.

The CB2 receptor was discovered and successfully cloned in 1993, and unlike CB1, it is primarily localized on immune cells and low expressed in the CNS [35,36]. Accumulated evidence has demonstrated the presence, although in low amount, of CB2 receptors in several peripheral tissues, including the GI tract, cardiovascular system, adipose tissue, liver, bone and the reproductive system [35].

At the cellular level, both the CB1 and CB2 receptors are localized in the cell membranes, although an intracellular localization has been reported [37]. Activation of cannabinoid receptors induces: (1) Inhibition of adenylyl cyclase activity, calcium channels and D-type potassium channels, (2) increase in the phosphorylation of mitogen-activated protein kinases, and (3) activation of A-type potassium channels [37].

**Figure 3:**
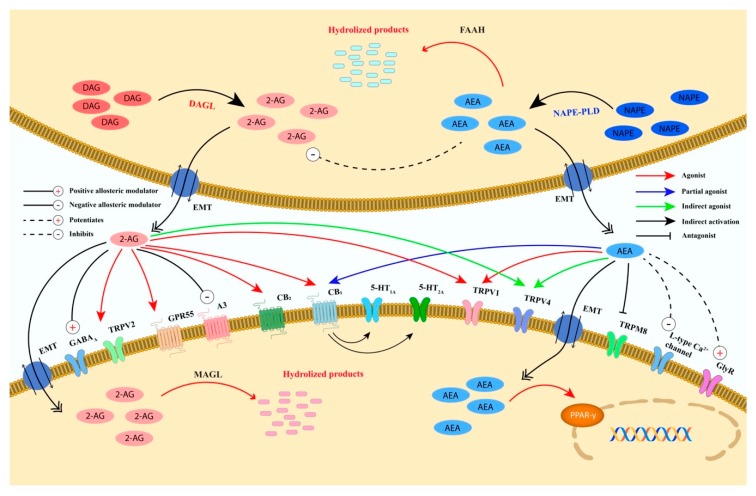
Schematic representation of the biosynthesis, degradation, and receptors’ binding of AEA and 2-AG. Anandamide and 2-AG are postsynaptically biosynthesized from the membrane’s phospholipids and degraded with different pathways and enzymes. AEA is mainly synthesized from NAPE by NAPE-PLD, whereas 2-AG is biosynthesized from DAG by DAGL-α and DAGL-β. The degradation of AEA is catalyzed by FAAH that is mainly expressed postsynaptically. 2-AG is degraded by MAGL that is expressed presynaptically and by two hydrolases named ABHD6 and ABHD12, expressed postsynaptically. Furthermore, AEA and 2-AG catabolism might also occur by the activity of other enzymes (e.g., NAAA, COX-2, and several LOX isoenzymes). AEA and 2-AG retrogradely activate presynaptic CB1. AEA is almost inactive on CB2, whereas 2-AG acts as a full agonist. In addition, AEA, directly or indirectly, also modulates the receptors/channels CB1, CB2, TRPV1 (at postsynaptic and presynaptic level), TRPV4, TRPM8, PPARγ, 5-HT_1A_, 5HT_2A_, L-type Ca^2+^channel, GlyR and negatively regulates 2-AG biosynthesis. 2-AG, directly or indirectly, modulates the receptors/channels TRPV1, TRPV2, TRPV4, GPR55, A3 adenosine, GABA_A_, 5-HT_1A_, and 5HT_2A_. The activation of CB1 by 2-AG suppresses either GABA or glutamate release. Abbreviations: ABDH6/12, αβ-hydrolase domain 6/12; AEA, anandamide; 2-AG, 2-arachidonoylglycerol; CB1, cannabinoid receptor 1; CB2, cannabinoid receptor 2; COX-2, cyclooxygenase-2; DAG, diacylglycerol; DAGL-α and DAGL-β, diacylglycerol lipase-α and β isoforms; EMT, endocannabinoid membrane transporter; FAAH, fatty acid amide hydrolase; GABA, γ-aminobutyric acid; GABA_A_, γ-aminobutyric acid type A receptor; GlyR, glycine receptor; GPR55, G protein-coupled receptor 55; 5-HT_1A_, 5-hydroxytryptamine 1A receptor; 5-HT_2A_, 5-hydroxytryptamine 2A receptor; LOX, lipoxygenase; MAGL, monoacylglycerol lipase; NAAA, N-acylethanolamine hydrolyzing acid amidase; NAPE-PLD, N-acyl-phosphatidylethanolamine-specific phospholipase D; PPARγ, peroxisome proliferator-activated receptor type-γ; TRPM8, transient receptor potential cation channel subfamily M member 8; TRPV2, transient receptor potential cation channel subfamily V member 2; TRPV4, transient receptor potential cation channel subfamily V member 4; TRPV1, transient receptor potential vanilloid type-1 channel.

The discovery of the cannabinoids receptors has led to the identification of endogenous ligands, called endocannabinoids, including anandamide (arachidonylethanolamide, AEA), 2-arachidonoylglycerol (2-AG), *N*-arachidonoyldopamine (NADA), noladin ether (2-arachidonoylglycerylether, 2-AGE) and virodhamine (O-arachidonoylethanolamine, O-AEA) [26]. However, until now, only AEA and 2-AG have been deeply studied. Although both endocannabinoids are synthetized on demand from the cell membrane phospholipids, they are synthesized, transported and inactivated differently (Figure 3) and possess a different affinity for the two cannabinoid receptors [26].

AEA is biosynthesized from N-acyl-phosphatidylethanolamine (NAPE) by NAPE-specific phospholipase D (NAPE-PLD) whereas 2-AG is synthesized from diacylglycerol (DAG) by DAG lipase (DAGL) α or β [26]. AEA and 2-AG are mainly inactivated by two enzymes, fatty acid amide hydrolase (FAAH) and monoacylglycerol lipase (MAGL), although other degradation enzymes/pathways have been identified [i.e., α/β -hydrolase domain containing (ABHD) 6 and 12 (for 2-AG), N-acylethanolamine acid amide hydrolase (NAAA) (for AEA), cyclooxygenase-2 (COX-2), 12-lipooxygenase (12-LOX), 15-LOX (for AEA and 2-AG)] [38]. Specifically, AEA is mainly hydrolyzed by FAAH into free arachidonic acid and ethanolamine, whereas MAGL catalyzes the degradation of 2-AG into arachidonic acid and glycerol (Figure 3).

AEA has been demonstrated to have both a high affinity for the CB1 receptor, where it acts as a partial agonist, and a low CB2 affinity [26]. Conversely, 2-AG has a moderate affinity for both receptors where it behaves as a full agonist [26].

In addition to CB1 and CB2 receptors, endocannabinoids have also been reported to modulate several other receptors and channels including transient receptor potential (TRP) channels and the G protein-coupled receptor (GPR) 55, GPR18, GPR119, γ-aminobutyric acid (GABA) A and glycine receptors [26] (Figure 3).

## 4. *Cannabis sativa* and its Phytocannabinoids

Considering the content of THC of *Cannabis sativa*, this species can be divided into two subspecies: *C. sativa* subsp. *Sativa* (THC < 0.3%) and *C. sativa* subsp. *Indica* (THC > 0.3%) [39]. *C. sativa* and *C. indica* can also be separated by morphology (*C. sativa* is taller with a fibrous stem, whereas *C. indica* is shorter with a woody stem, Figure 1), and by differences in their original geographic range (*C. sativa* in Europe and *C. indica* in Asia) [4].

### Psychoactive and Non-Psychoactive Phytocannabinoids Molecular Targets

As mentioned above, more than 120 phytocannabinoids have been identified in the plant *Cannabis sativa*. Among these, the most studied are THC, CBD, CBG, CBC and CBDV (Table 2). These substances are present in the plant as their carboxylated version (i.e., tetrahydrocannabinolic acid (THCA), cannabidiolic acid (CBDA), cannabigerolic acid (CBGA), etc.). During the harvest, drying, and storage of *Cannabis sativa* or when heat via smoking, the carboxylated phytocannabinoids are decarboxylated, and therefore, converted into the corresponding chemically neutral version [40,41].

THC, isolated in 1964, is the most abundant psychoactive cannabinoid in *Cannabis sativa*. In addition to the psychotropic effects (due to the activation of the CNS CB1 receptor), THC by acting on CNS CB1: (1) Induces drowsiness and impairment of short term memory; (2) reduces alertness, the accuracy of psychomotor task performance, motor coordination, muscle tone and pain perception; (3) exerts antinauseant and antiemetic effects; and (4) increases appetite [59,79]. Moreover, at low doses, THC induces mild euphoria, relaxation and reduces anxiety, whereas, at high doses, leads to dysphoria, sensory distortion, hallucinations, and increases anxiety. THC also exerts other numerous pharmacological effects that are mediated by either CB1 and/or CB2 receptor activation or other targets. Specifically, THC induces tachycardia, antispasticity and anti-convulsant effects, bronchodilatation, bronchial irritation, and ocular hypotonicity. In addition to CB1 and CB2 activation, THC can act as an agonist of the receptors/channels GPR55, GPR18, peroxisome proliferator-activated receptor gamma (PPARγ), transient receptor potential (TRP) A1, TRPV2, TRPV3 and TRPV4, and as an antagonist of the receptors/channels TRPM8 and 5HT3A (see Table 2 for references). Moreover, THC is able to modulate orthosteric agonist affinity and/or efficacy of µ/δ opioid receptors (negative allosteric modulator) and GlyRα1/α3 receptors (positive allosteric modulator) [52,53,54]. Finally, this psychoactive phytocannabinoid is also able to increase AEA (inhibiting its uptake by competing with AEA for binding to fatty acid binding proteins, FABPs) and adenosine levels [57,58].

For its pharmacological effects, THC has been used to treat nausea and vomiting associated with cancer chemotherapy and to stimulate appetite in patients with acquired immunodeficiency syndrome (AIDS). THC has also been used to treat pain, spasticity in patients with multiple sclerosis, and glaucoma. However, the clinical use of THC is limited today, due to its psychoactive effects. Thus, in the last forty years, much attention has been paid to non-psychoactive phytocannabinoids, such as CBD, CBG, CBC, and CBDV.

Although CBD was isolated from marijuana in 1940, interest in this compound started when it was shown that CBD was able to reduce the psychotropic effect of Δ^9^-THC [80,81]. Subsequently, it has been demonstrated that CBD possesses a wide spectrum of actions including neuroprotective, analgesic, anti-inflammatory, anti-epileptic, anxiolytic and antipsychotic effects [82,83]. Moreover, CBD improves sleep disorders and induces mood stabilization [84,85,86]. CBD effects are mediated by its action on multiple molecular targets [83,87]. CBD has been demonstrated to have a low affinity for both CB1 and CB2 receptors where mainly acts as a negative allosteric modulator. CBD acts as an agonist of the receptors/channels TRPA1, TRPV1, TRPV2, TRPV3, PPARγ, 5-HT1A, and A1A, and as an antagonist of the receptors GPR55, GPR18, and 5HT3A (see Table 2 for references). CBD is an inverse agonist of the receptors GPR3, GPR6, and GPR12 [63,64]. CBD is an allosteric modulator of μ- and ∂-opioid receptors [52,53], GABAA [69] and GlyRα1/α3 receptors [71]. CBD functions as an anandamide reuptake and breakdown inhibitor by targeting FABPs [57]. Finally, recently it has been demonstrated that, in addition to the negative allosteric effect on the CB1 receptor, CBD affects the Δ^9^-THC cognitive impairment in an adenosine A2A receptor (A2AR)-dependent manner [88].

Clinical studies have demonstrated that CBD has a good safety profile, and it is effective in the drug-resistant seizure in patients with severe rare forms of epilepsy [89]. In June 2018, CBD was approved by the Food and Drug Administration (FDA), with the trade name of Epidiolex, as an anti-epileptic drug for patients (2 years of age and older) with Dravet or Lennox-Gastaut syndromes. By contrast, clinical evidence regarding the efficacy of CBD in psychiatric disorders is preliminary. Moreover, encouraging preliminary evidence also exists on the efficacy of CBD in patients with autism [90]. Specifically, CBD seems able to reduce aggression, anxiety and hyperactivity in patients with autism. In several countries, Sativex^®^, an oromucosal spray containing an equimolecular combination of Δ^9^-tetrahydrocannabinol-botanical drug substance (Δ^9^-THC-BDS) and cannabidiol-botanical drug substance (CBD-BDS), has been approved for the use of neuropathic pain, due to multiple sclerosis.

Another abundant non-psychotropic cannabinoid in *Cannabis sativa* is CBG. Pharmacodynamic studies have shown that CBG targets components of the ECS, as well as a range of other receptors and ion channels. CBG displays a weak affinity for CB1 and CB2 receptors where it acts as a partial agonist. In addition, CBG acts as an agonist of α2-adrenoceptor, TRPA1, TRPV1, and TRPV2 receptors, and as an antagonist of TRPM8, TRPV4 and 5HT1A receptors (see Table 2 for references). Moreover, CBG, as CBD, inhibits AEA uptake, MAGL and lysophosphatidylinositol (LPI)-induced GPR55 signaling (see Table 2 for references).

CBC, discovered in 1966, acts as an agonist of CB2 receptors, TRPA1 (it seems to be the most potent TRPA1 agonist among phytocannabinoids), TRPV3, TRPV4 channels and an antagonist of TRPM8 channels (see Table 2 for references). Similar to CBG, CBC increases AEA (inhibiting its uptake) and MAGL levels (see Table 2 for references).

CBDV has been reported to have a low affinity for CB1 and CB2 receptors. CBDV acts as an agonist of TRPA1, TRPV1, TRPV2, TRPV3 and TRPV4 channels, and as an antagonist of TRPM8 channels (see Table 2 for references). Moreover, CBDV inhibits AEA uptake, DAGL and LPI-induced activation of GPR55. CBDV exert anti-convulsant [91], neuroprotective properties [92], and rescues cognitive deficits [93]. Today, CBDV is under clinical investigation for epilepsy treatment and autism spectrum disorders (ClinicalTrials.gov Identifier: NCT02369471, NCT03202303) [94].

## 5. Cannabidiol and Other Non-Psychoactive Phytocannabinoids for Gastrointestinal Health

Receptors and ligands of the ECS are widely expressed in relevant areas and systems, both within and outside the GI tract, related to the control of GI functions. As mentioned above, all the components of the ECS are present within the GI tract [95,96,97,98]. Consistent with these data, numerous reports have identified an important role for cannabinoids, including non-psychoactive phytocannabinoids, in the control of GI functions in health and disease states. Available experimental data show that the ECS is implicated in the control of motility, secretion, epithelial barrier function and viscerosensitivity, being a key component in the maintenance of GI homeostasis and a significant player in several pathophysiological states implicating a neuro-immuno-endocrine dysregulation of the GI tract (Table 3) [96,97,99,100,101,102,103,104,105,106,107,108].

Within the GI tract, the ECS has, overall, an inhibitory role. Indeed, direct or indirect activation of cannabinoid receptors reduces gastric and intestinal motility, and several motility-related parameters [97,99,106,107,109,110,111]; inhibits gastric and intestinal secretion [97,101,112]; modulates visceral sensitivity, reducing pain sensations [97,100,104,111,113], and reduces the activity of the local immune system, lowering inflammatory responses [97,101,114].

Interestingly, although the effects of the ECS can be observed under physiological conditions, it is under pathophysiological states when the system probably displays its full significance. Under pathophysiological conditions, many of the components of the ECS are upregulated. In particular, increased expression of cannabinoid receptors and/or enhanced endocannabinoid levels, due to changes in the expression and activity of the enzymatic machinery implicated in the synthesis and metabolism of endocannabionids, have been observed (see also Table 2) [95,114]. Therefore, the system, as a whole, displays higher activity, including the manifestation of modulatory/regulatory functions not evidenced under physiological conditions [95,114]. Taking into account these observations, the potential therapeutic use of cannabinoids for the treatment of GI-related disorders have been widely explored [97,99,100,101,105,106]. However, the central psychoactive effects (such as catalepsy, hypothermia, hyperphagia, anxiety, panic attacks, paranoia and cognitive impairment) of the main active component of *Cannabis sativa*, THC, has limited its clinical use [115]. In this context, non-psychotropic phytocannabinoids, with similar pharmacological properties as THC, but devoid of the undesired central psychoactive effects, used as classical drugs or as herbal nutraceuticals, might represent an interesting alternative [116,117]. Here, we will focus on recent evidence related to the effects and use of CBD, and other non-psychoactive phytocannabinoids, for the prevention and treatment of GI diseases.

### 5.1. Role in Irritable Bowel Syndrome

Irritable bowel syndrome (IBS) is a multifactorial, highly prevalent functional GI disorder (10–20% in the developed world), characterized by the presence of functional alterations in the absence of evident underlying organic causes. Main alterations observed include GI motility disturbances [with predominant constipation (IBS-C), predominant diarrhea (IBS-D) or mixed bowel habits (IBS-M)], abnormal visceral sensitivity (hypersensitivity), altered epithelial barrier function (leaky gut), dysbiosis and disruptions of the brain-gut axis leading, mainly, to abnormalities in the processing of visceral afferent inputs [118,119,120,121,122]. Because of these characteristics, modulation of the ECS might be beneficial for IBS patients. Overall, cannabinoid-based therapies for functional GI disorders are focused predominantly on pain relief and modulation of intestinal motility.

Early reports indicated that CBD did not affect intestinal motility (gastric emptying, small intestinal transit and defecation) when tested in normal, healthy, animals, compared with other natural cannabinoids (such as Δ^8^-THC or Δ^9^-THC) that elicited inhibitory effects [123,124,125,126,127]. These observations were further confirmed using pure CBD and a cannabis extract with high content in CBD. While pure CBD had no effects on healthy animals, extracts with high content in CBD had weak inhibitory effects, likely due to the presence of other bioactive substances (such as THC and THC-related molecules) [128]. Similar lack of effects was also observed, in physiological conditions, for the non-psychoactive phytocannabinoid CBC [126], while cannabinol (CBN) showed only weak inhibitory effects on GI transit compared to other cannabinoids [129,130]. However, when tested in states of altered motility, particularly in states of hypermotility, either CBD, CBN or CBC exhibited significant modulatory effects. For instance, in the model of croton oil-induced intestinal irritation in mice, which occurs with increased intestinal motility (i.e., a reduction in transit time), CBN [130], CBD [127,131] and CBC [126] completely normalized intestinal transit. Interestingly, similar positive effects were also observed for CBD in lipopolysaccharide (LPS)-induced intestinal inflammation and hypomotility [132]. Altogether, these observations agree with the view that the ECS exhibits its full activity not in physiological conditions but under pathophysiological states [95,114]. Moreover, they indicate a potential for non-psychotropic phytocannabinoids, at least as it relates to CBD, CBN or CBC, on the treatment of IBS-associated dysmotility, particularly for patients with the diarrhea predominant (IBS-D) or the mixed bowel habits (IBS-M) forms of the disease, as already suggested for the psychoactive cannabinoid dronabinol [133,134]. Because of the inflammatory component associated with some of the animal models used (LPS- and croton oil-induced dysmotility), these observations, although discussed here, might also bear interest for inflammatory disorders of the gut (see Section 5.2).

Abdominal pain associated with the sensitization of visceral sensory afferents and/or the derangement of sensory processing along the brain-gut axis is a key component of IBS [120]. Preclinical evidence, based on the colorectal distension model in rats and mice and the writhing test in mice, indicated that cannabinoid receptors, located in the CNS and/or at peripheral sites, may be involved in visceral pain and the development of visceral hypersensitivity [111,113,135,136,137,138,139,140,141,142]. Of note, the antinociceptive effects of cannabinoids were enhanced during inflammation [136,137], further supporting a role for the ECS in the development of visceral hypersensitivity and the therapeutic potential for the treatment of abdominal/visceral pain in IBS and other gut disorders, such as chronic constipation or inflammatory disorders. At a pre-clinical level, so far, only two studies have addressed the efficacy of phytocannabinoids modulating visceral pain, with some conflicting results. An early report showed that the non-psychotropic phytocannabinoids CBD and CBG showed significant analgesic activity in the writhing test in mice, although with lower efficacy than the psychoactive Δ^1^-THC [135]. On the other hand, CBN was inactive [135]. Lately, also using the writhing test model in mice, Booker et al. showed that only THC and CBN were able to elicit antinociceptive effects, through a CB1-dependent mechanism [138]. However, CBN exhibited a significantly lower potency than THC, per the differences in their relative binding affinity for CB1 receptors (K_i_; THC: 47.7 nM; CBN: 129.3 nM) [138]. Besides CBN, other non-psychotropic phytocannabinoids tested, namely CBD and CBC, did not manifest any analgesic effect in this model [138]. Although with some discrepancies, these reports suggest a potential therapeutic value for CBN, CBD, and CBG modulating visceral nociception. However, more research is needed to determine their effects in other visceral pain models (namely colorectal distension).

Despite the promising preclinical data, the efficacy of cannabinoids modulating IBS-associated abdominal pain has been difficult to prove in a clinical context, at least as it relates to the use of tetrahydrocannabinols [THC and dronabinol] [133,143]. This is not exclusive to IBS, since a lack of modulatory effects was also observed in patients with chronic abdominal pain [144] or post-operative abdominal pain [145]. On the other hand, no clinical studies addressing the modulation of visceral pain have been performed with non-psychotropic phytocannabinoids. According to the clinical trials register (https://clinicaltrials.gov; accessed 03/22/2020), a trial was started in 2016 assessing the effects of a CBD-containing chewing gum (CanChew^®^) on IBS, having as a primary outcome changes in pain (ClinicalTrials.gov Identifier: NCT03003260). However, no additional information is available. Overall, this indicates that cannabinoids, per se, may be of little usefulness to reduce visceral hypersensitivity in IBS patients (or other GI conditions presenting abdominal/visceral pain). However, combinations with other pain modulatory agents, such as opioids, is regarded as an interesting alternative approach.

### 5.2. Role in Inflammatory Bowel Disease

Inflammatory bowel diseases (IBD), including Crohn’s disease (CD) and ulcerative colitis (UC), is a multifactorial, chronic immune-mediated disease of the GI tract that results from a complex interaction between environmental, genetic and epigenetic risk factors that cause an inappropriate mucosal immune response leading to intestinal inflammation. IBD is characterized by periods of inflammatory flares, quiescence, and relapse, which places a substantial psychologic, emotional, and symptomatic burden on affected individuals [146,147]. The current therapeutic goals in managing IBD are reduction of inflammation, elimination of symptoms (mainly abdominal pain, fecal bleeding, diarrhea and weight loss), improvement in quality of life, and the prevention of complications [146,147]. Although the pathophysiology of the disease is not completely understood, knowledge of some of the underlying immunopathological mechanisms has led to the development of several effective therapies, used in the induction and maintenance of remission of disease activity [148,149]. Current therapies, however, are not effective in all patients, and patients that do respond often lose response over time; moreover, in many cases, symptoms persist even when inflammation is controlled and in remission. In addition, some patients develop adverse events that necessitate treatment discontinuation [150]. Therefore, IBD patients often turn to complementary medications, including various forms of cannabis, to combat symptoms related to their disease [2,151,152].

Patients have reported using cannabis to relieve symptoms of abdominal pain, nausea, diarrhea and anorexia, as well as to improve mood and quality of life [152,153,154,155]. However, the evidence supporting the efficacy of cannabis in IBD is limited, and clinical guidelines and recommendations to assist physicians remain inadequate. Moreover, the long-term safety profile of cannabis in patients with IBD has not been established and remains a matter of concern [151,152]. In this context, non-psychotropic phytocannabinoids may be a valid and safe adjunct to medications in controlling inflammation, as well as improving symptomatology and quality of life. Therapeutic use of non-psychotropic phytocannabinoids in IBD is justified by their multiple modulatory roles within the GI tract, particularly affecting the activity of the local immune system (affecting cytokine, immunoglobulin production, and immune cell migration), but also because of their effects on motility and sensation (as discussed above for IBS) [156].

The immunomodulatory and anti-inflammatory actions of cannabinoids have been demonstrated in several experimental rodent models of intestinal inflammation [101,106,114,157,158]. Briefly, preclinical evidence shows: (1) Immunosuppressive effects derived from the impairment of both cellular and humoral immunity by reducing inflammatory cell recruitment, inducing T cell apoptosis and suppressing the production of numerous pro-inflammatory cytokines and chemokines [101,159]; (2) an increase in wound healing, leading to the restoration of intestinal epithelial barrier function [160]; (3) inhibition of GI motility and secretion leading to the reduction of diarrhea [99,110,129,130,161,162] (see also comments related to IBS); and (4) a reduction of visceral hypersensitivity and abdominal pain (see comments related to IBS). In general, these actions inhibited the development of colitis and reduced the already established inflammation.

As it relates, specifically, to non-psychotropic phytocannabinoids, most of the studies performed have explored the anti-inflammatory actions of CBD [156], with only a few studies assessing the effects of other compounds, namely CBG, CBC, and CBDV (see Table 4). The anti-inflammatory effects of other non-psychotropic phytocannabinoids have not been studied so far.

Cannabidiol shows potent anti-inflammatory actions in different models of intestinal inflammation in mice and rats (dinitrobenzene sulfonic acid- [DNBS-], trinitrobenzene sulfonic acid- [TNBS-] and LPS-induced inflammation). In general, local or systemic administration of CBD resulted in a dose-related amelioration of disease parameters, reduction of structural damage and contention of inflammation-associated up-regulation of different cytokines, chemokines and markers of oxidative stress [131,132,156,163,164,165,166]. Moreover, some of these effects were also reproduced in in vitro conditions. For instance, CBD reduced the production of reactive oxygen species (ROS) and lipid peroxidation in Caco-2 cells cultures [166], and counteracted LPS/interferon gamma (IFNγ)-induced inflammatory-like responses in cultured human-derived colonic biopsies of UC patients [163]. Similar to that reported for CBD, CBG and CBC showed significant anti-inflammatory activity during DNBS-induced colitis in mice [167,168]. Cannabigerol reduced disease scores, attenuated alterations associated with oxidative stress and normalized cytokines expression [167]. Furthermore, in in vitro conditions, CBG also reduced nitric oxide (NO) production in macrophages and reduced the formation of ROS in intestinal epithelial cells [167]. Similar effects were observed for CBC, which ameliorated DNBS-induced colonic inflammation and restored epithelial barrier function [168]. Additionally, CBC reduced LPS-induced activation of murine peritoneal macrophages in vitro, an effect likely contributing to its in vivo anti-inflammatory activity [168]. Lastly, a recent report has evaluated the anti-inflammatory effects of CBDV, a compound under evaluation for the treatment of autism spectrum disorder and epilepsy/focal seizures [94,169], thus supporting its therapeutic potential. Oral CBDV reduced colonic inflammation (structural damage, inflammatory infiltrate, altered permeability and cytokine production), in the DNBS-induced colitis model in mice [128]. CBDV also reduced cytokine expression in colonic biopsies from pediatric patients with UC, which also supports the translational value of these observations [128].

The mechanisms by which cannabinoids exert their anti-inflammatory effects are still a matter of debate. Although cannabinoid receptors are expressed in relevant immune-related sites important in inflammatory responses, cannabinoids, particularly phytocannabinoids, also show activity on non-cannabinoid receptors known to participate in inflammatory mechanisms (see above). For instance, several non-psychoactive phytocannabinoids, such as CBN, CBD, CBC, CBDV, and CBG, act as agonists and desensitizers of different TRP channels [56,170], known to participate in inflammatory responses and to be specifically regulated in IBD [171,172,173]. Additionally, indirect effects on cannabinoid receptors through the inhibition of endocannabinoid metabolism or uptake have also been described. For example, CBD, besides its effects on cannabinoid receptors, activate/desensitize a variety of TRP channels, including TRPV1, 2, 3 and ankyrin 1-type (TRPA1) [49,51]. Cannabidivarin shows very little affinity for cannabinoid receptors, but it is an inhibitor of endocannabinoid cellular reuptake [174], a weak inhibitor of MAGL (the main enzyme involved in the inactivation of the endocannabinoid 2-AG) and a potent activator of TRPA1 channels [49,51,56]. Therefore, part of the anti-inflammatory effects of the phytocannabinoids of interest here might be related to non-cannabinoid receptors-mediated effects or to reflect a modulation of the endocannabinoid tone/activity, thus avoiding some of the safety issues associated with the use of other cannabinoids.

Despite the relatively extensive pre-clinical evidence, only two studies have assessed, so far, the efficacy of non-psychoactive phytocannabinoids in a clinical setting, and these have addressed only the use of CBD (pure or a CBD rich botanical extract). The only study assessing the efficacy of CBD in CD was negative, with no improvement in disease activity as measured by a CD Activity Index (CDAI), as well as several laboratory parameters [175]. Interestingly, the treatment was safe, with no differences to placebo in adverse effects. As the authors discussed, the negative results might be due to the small number of cases included (19), the low dose tested (10 mg, orally) or the lack of the necessary synergism with other cannabinoids [175]. An additional proof-of-concept study addressed the effects of a CBD-rich botanical extract in UC [176]. The main result of this study was the lack of tolerability of the botanical extract, with 90% of patients reporting treatment-related adverse effects as compared with 48% receiving placebo. There was no difference in the primary endpoint of clinical remission between groups. However, there was a trend toward improved quality of life scores and improvement in patients’ global impression of change based on the per-protocol analysis. The authors suggest that the CBD-rich botanical extract may have provided therapeutic benefit to those patients who tolerated it; and they encourage future studies, reviewing the formulation, titration, and dosing, to improve tolerability [176]. Overall, a recent meta-analysis that reviewed the evidence of cannabis and CBD on UC and CD was unable to make any firm conclusions on their safety or efficacy in IBD [177,178]. These reports concluded that further studies with a larger number of patients, different doses and routes of administration and a follow-up to assess the long-term safety outcomes are still necessary. Moreover, the potential use as nutraceuticals of CBD and other non-psychotropic phytocannabinoids should also be considered for clinical studies.

### 5.3. Role in Gastrointestinal Cancer

GI cancers, and particularly colorectal cancer (CRC), are among the most frequent in the general population [179,180]. Cannabinoids have been used historically to alleviate several cancer-related symptoms, such as pain, emesis, cachexia or dysgeusia, with the general objective of increasing patients’ quality of life [181,182,183]. Significant changes in the ECS have been described in CRC. In particular, increased endocannabinoid levels, down-regulation of CB1 receptors and up-regulation of CB2 receptors have been observed in intestinal specimens of CRC patients [184,185]. These observations support the potential role of cannabinoids regulating cancer progression. The effects of cannabinoids on intestinal carcinogenesis have been evaluated in CRC epithelial cells, in experimental models of colon cancer and on gastric cancer cell lines [95,106,186,187]. Overall, cannabinoids might exert protective effects on carcinogenesis directly, through activation of cannabinoid receptors, or indirectly, through elevation of endocannabinoid levels via inhibition of metabolizing enzymes (particularly FAAH). In any case, activation of cannabinoid receptors is associated with anti-proliferative effects, the promotion of apoptosis, the inhibition of tumor cells migration and/or the inhibition of angiogenesis.

As it relates to non-psychoactive phytocannabinoids, the studies performed have centered on the potential anticancer use of CBD [188,189]. Interest in CBD is supported by evidence of its potential therapeutic value. First, its main mechanism of action (elevation of endocannabinoid levels by inhibition of enzymatic degradation) and pharmacological effects (antioxidant and anti-inflammatory) [189]. Secondly, CBD is known to have antitumor activity against Noxa activation, inhibition of mTOR/cyclin D1, and G-protein-coupled receptors/mitogen-activated protein kinase (GPR/MAPK) pathway in various cancers, such as pancreatic [190], glioblastoma [191], leukemia [192], and breast cancer [193]. Thirdly, GPR55 is implicated in the migratory behavior of HCT116 colon cancer cells and seems to play an important role in the prevention of metastasis [194]. Finally, the synthetic CBD analog, O-1602, has been shown to decrease viability and induce apoptosis in colon cancer cells and to reduce tumor area and tumor incidence in a colitis-associated colon cancer mouse model [195]. Altogether, this evidence suggests that CBD might have beneficial effects on GI cancers.

Multiple mechanisms are likely to be involved in CBD anti-proliferative effects in in vitro conditions, likely depending upon the CRC cell lines used. Early studies in SW40 colon cancer cells showed that CBD induces phosphatases and phosphatase-dependent apoptosis [196]. Subsequent studies in Caco-2 and HCT116 cells, showed that CBD protected DNA from oxidative damage, increased endocannabinoid levels and reduced cell proliferation in a CB1-, TRPV1- and PPARγ-antagonists sensitive manner [197]. In HCT116, colo205 and DLD-1 tumor cells, apoptosis in a Noxa-and-ROS-dependent manner was observed upon exposure to CBD [198,199]. Interestingly, no toxic/anti-proliferative effects were observed in healthy colonic cells [199,200], thus reinforcing the favorable safety profile of CBD. Consistent with these in vitro observations, anti-cancer effects of CBD have also been demonstrated in vivo in different murine models of CRC. In a model of azoxymethane-induced colon tumorigenesis in mice, a CBD-enriched botanical extract or pure CBD reduced preneoplastic lesion, polyps and tumor formation and counteracted some of the molecular changes associated with the process (up-regulation of phospho-Akt and down-regulation of caspase-3) [197,200]. Lastly, a recent study investigated the effect of CBD on the CT26 colon cancer line, in vivo, in an animal model showing encouraging effects on reducing colon cancer growth and decreasing tumor size, likely due to an increase of the antioxidant enzymes SOD and glutathione peroxidase (GPX) [201].

Similar effects to those observed for CBD in CRC cell lines have also been reported for gastric carcinoma cells. In human gastric cancer cells SGC-7901, CBD inhibited proliferation and colony formation through the induction of cell cycle arrest at the G0-G1 phase and apoptosis by increasing ROS [202]. Moreover, CBD promoted apoptosis in several gastric cancer cell lines (AGS, MKN45, SNU638, and NCI-N87) by X-linked inhibitor apoptosis (XIAP)/Smac-dependent mechanisms and mitochondrial dysfunction [203]. Interestingly, and as also reported for normal colonic cells, gastric normal epithelial HFE-145 cells were not affected [203].

Regarding other non-psychoactive phytocannabinoids, the information available is very limited. A report has assessed the anticancer effects of CBG on colon carcinogenesis, with similar general effects and mechanisms of action as those described for CBD. Cannabigerol inhibited the growth of CRC cells (Caco-2 and HTC 116) mainly *via* a pro-apoptotic mechanism, associated with ROS overproduction, and hindered the development and the growth of colon carcinogenesis in vivo (azoxymethane-induced colon tumorigenesis and HCT 116 CRC xenograft model in mice) [200]. An ethanol extract of *Cannabis sativa* enriched in CBGA showed low cytotoxic activity on colon cancer cells and a positive interaction with a THCA-enriched extract, which resulted in enhanced cytotoxic activity [204]. However, the interest of CBGA might be limited because of its low intrinsic activity and the fact that it did not show a positive interaction with other non-psychotropic phytocannabinoids (mainly CBD) on leukemic cells [192].

As it related to other GI cancers, the CB1 receptor is overexpressed in esophageal squamous carcinoma cell lines, and CB1 receptor activation appeared to promote cell proliferation and invasion [205]. Thus, a potential therapeutic utility in the treatment of esophageal cancer must be considered.

### 5.4. Other Disorders and Diseases of the Gastrointestinal System

#### 5.4.1. Nausea and Emesis

*Cannabis sativa* plant has been used for several centuries for the attenuation of nausea and vomiting. Lately, the use of cannabinoids has been considered, particularly, for the control of symptoms of nausea and anticipatory nausea in chemotherapy patients, which are less well controlled by the current conventional therapies. The main drawback for their clinical use is their psychoactive effects. Therefore, non-psychoactive phytocannabinoids offer a safe alternative to other cannabinoids.

Preclinical research indicates that CBD may be clinically effective for treating both nausea and vomiting produced by chemotherapy or other therapeutic treatments. However, the clinical effectiveness of this compound in reducing nausea and vomiting has not been evaluated. Nevertheless, positive effects have been observed when combined with THC [206], and this combination (a mixture of THC and CBD in a ratio of approximately 1:1, together with small amounts of other cannabinoid derivatives; Sativex^®^) is under further clinical investigation [207]. Cannabidiol, within a limited dose range, suppresses nausea and vomiting in different animal models by a non-cannabinoid receptor-dependent mechanism [99,208,209]. Indeed, the anti-nausea/anti-emetic effects of CBD may be mediated by indirect activation of somatodendritic 5-HT_1A_ receptors in the dorsal raphe nucleus; activation of these autoreceptors reduces the release of 5-HT in terminal forebrain regions [66,208]. However, some paradoxical effects of CBD on emesis have been described. Indeed, CBD seems to have a biphasic effect on emesis, with high and low doses potentiating and inhibiting, respectively, toxin-induced vomiting and anticipatory retching in *S. murinus* [208]. Of note, CBG, because of its antagonistic pharmacological activity at both CB1 and 5-HT_1A_ receptors, reverses the anti-emetic actions of low-dose CBD [210]. Moreover, the pro-emetic properties of CBD (at higher doses) and CBG may play a role in severe nausea and vomiting observed in patients with cannabinoid hyperemesis syndrome [211]. The cannabinoid hyperemesis syndrome (CHS) is a distinct syndrome, related to cyclic vomiting syndrome, characterized by recurrent vomiting associated with abdominal pain, hypothermia and compulsive hot bathing in the setting of chronic cannabinoid use [212,213]. Although the causes remain unknown, it has been associated with the potency of the THC consumed, the amount of use and the duration of use [213]. High levels of circulating THC might induce gastric stasis and delayed gastric emptying, contributing to the induction of nausea and vomiting, as suggested from animal studies assessing the effects of the synthetic cannabinoid WIN 55,212-2 on gastric motility [214,215]. Although interactions between the multiple phytocannabinoids present in *Cannabis sativa* have to be taken into consideration, the relevant role that THC seems to play suggests that its substitution (or at least reduction in concentration) by other phytocannabinoids might be beneficial in avoiding CHS. Therefore, the selective use of non-psychoactive phytocannabinoids might contribute to reducing the risk of CHS as a potential undesired side effect of chronic cannabinoid use.

#### 5.4.2. Gastric Secretion and Gastroprotection

Overall, cannabinoids inhibit gastric secretion through CB1-dependent mechanisms that might implicate a modulation of vagal activity and a direct inhibitory effect of parietal cells [95,101]. CB1-dependent mechanisms also mediate gastroprotective effects of cannabinoids, as shown in different experimental models of gastric erosion and ulcer formation [95,101]. Gastroprotective effects of cannabinoids involve multiple mechanisms, implicating central and peripheral effects, as well as acid secretory-dependent and independent responses [101]. No specific studies have been performed assessing the effects of non-psychoactive phytocannabinoids. However, taking into consideration the effects described and the implication of local (gastric) mechanisms, the use of these compounds as nutraceuticals in this context warrants further investigations.

Based on the anti-inflammatory properties of CBD [156], its therapeutic potential in the management of chemo- and radiotherapy-induced oral mucositis (which might bear similarities with gastric erosions and ulcers) has been assessed [216]. In a preclinical study in rats, CBD exerted an anti-inflammatory effect in the early phase of oral wound healing process although it was not sufficient to promote the clinical improvement of oral traumatic ulcerative lesions [217]. In any case, the clinical data available is not conclusive, and additional studies are required. Again, because of the desired local action, the administration of CBD, or other non-psychoactive phytocannabinoids, as nutraceuticals should be considered.

#### 5.4.3. Gastroesophageal Reflux Disease

The lower esophageal sphincter (LES), a specialized region of the esophageal circular smooth muscle, allows the passage of a swallowed bolus to the stomach and prevents the reflux of gastric contents into the esophagus. The cannabinoid system has been implicated in the mechanisms regulating LES relaxation [218]. In dogs and ferrets, cannabinoids inhibited transient LES relaxations through a CB1-dependent mechanism. These observations are of interest because transient LES relaxations are the predominant mechanism of gastroesophageal reflux disease [218]. In line with these preclinical observations, positive effects of THC have been observed in humans, although with issues related to the safety profile (central psychotropic activity) [219]. Besides the inhibition of transient LES relaxations, multiple pharmacological actions might contribute to the beneficial effects of cannabinoids in the treatment of gastroesophageal reflux disease: Inhibition of gastric acid secretion, reduction of microvascular leakage and bronchoconstriction associated with reflux and reduction of pain associated with esophageal hypersensitivity [218].

Studies performed so far have concentrated on the use of THC or different synthetic cannabinoids, without data available as it relates to non-psychoactive phytocannabinoids. Nevertheless, the current knowledge indicates that the use of non-psychoactive phytocannabinoids for the treatment of gastroesophageal reflux disease is worthy of exploring as an adjunct therapy with acid inhibition.

In summary, compelling evidence, both preclinical and clinical, supports the view that non-psychoactive phytocannabinoids have therapeutic potential for the treatment of several GI diseases and as promoters of GI health. Nevertheless, the clinical evidence is still limited, and additional studies are necessary, addressing both efficacy and safety. In particular, CBD has raised significant interest, mainly following its reschedule by the USA Drug Enforcement Administration (DEA) to the least restrictive Schedule V category, as of September 2018 (compared to cannabis, included in Schedule I category) [116]. In many instances, nutraceuticals are regarded as disease-preventive treatments. However, no clinical studies have been performed addressing this potential use. Nevertheless, preclinical data in animal models of intestinal inflammation suggest that phytocannabinoids might act both preventing the development of inflammation, as well as ameliorating an already established state of inflammation. This suggests, at least, a potential in the maintenance of the remission state and the prevention of disease flares in IBD patients.

## 6. Conclusions

Phytocannabinoids exert potent actions throughout the body. Data reported mainly from preclinical research, but also from the available clinical evidence, have demonstrated their high therapeutic potential to treat the diseased GI tract, from functional to organic pathologies. This not only applies to the well-known psychoactive compound, THC, but also to non-psychoactive molecules like CBD and others which have been more scarcely studied so far.

Interestingly, the non-psychoactive phytocannabinoids may be considered as nutraceuticals, and it is envisaged that they will soon find their place, not only in therapy, but in the food industry, leading to new formulations for a healthy life in general, and for a healthy GI tract function, in particular.

The eventual introduction and wide use of hemp-derived non-psychoactive phytocannabinoids as food ingredients will require clearer (and more flexible) regulations, based on clear, evidence-based, scientifically demonstrated, knowledge of their effects and mechanisms of action, which are urgently needed.

## Figures and Tables

**Table 1:** Different uses of hemp seed as a food ingredient. Data obtained from FoodData Central database.

Type of Ingredient	Type of Food	Number of Products
Hemp seed	Seeds	56
	Chocolate	13
	Cereal	80
	Bars and snacks	178
	Bakery products	37
	Beverage	52
	Dressings, butter	51
	Prepared food	24
	Yogurt, cheese, ice-cream (vegan)	13
Hemp protein extract	Beverage	78
	Bars and snacks	40
	Powder	24
	Capsules	1
Hemp oil	Oil	9
	Beverage	7
Hemp flour	Pasta	2
	Veggie burger or sausage	12
	Bakery products	3

**Table 2:** Molecular targets of the most abundant phytocannabinoids in *Cannabis sativa*.

**Δ^9^-TETRAHYDROCANNABINOL**		
**Chemical structure**	**Molecular Targets**	**References**
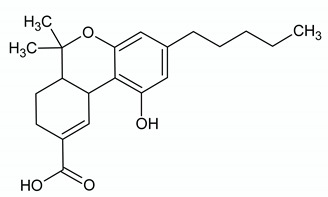	CB1 partial agonist	[42]
	CB2 partial agonist	[42]
	GPR55 agonist	[43]
	GPR18 agonist	[44,45]
	5HT_3A_ antagonist	[46]
	PPARγ agonist	[47,48]
	TRPA1 agonist	[49]
	TRPV2 agonist	[50]
	TRPV3 agonist	[51]
	TRPV4 agonist	[51]
	TRPM8 antagonist	[49]
	µ and δ opioid allosteric modulator	[52,53]
	GlyR α_1_ and α_3_ positive allosteric modulator	[54]
	AEA uptake inhibition by targeting FABPs	[55,56,57]
	Adenosine reuptake inhibitor	[58]
**CANNABIDIOL**		
**Chemical structure**	**Molecular Targets**	**References**
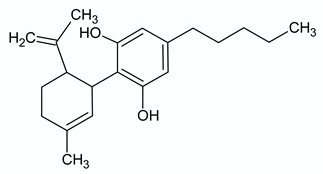	CB1 inverse agonist and negative allosteric modulator	[59,60]
	CB2 partial agonist and negative allosteric modulator	[59,61]
	GPR55 antagonist	[62]
	GPR18 antagonist	[44]
	GPR3 inverse agonist	[63]
	GPR6 inverse agonist	[63]
	GPR12 inverse agonist	[64]
	A_1A_agonist	[65]
	5HT1_A_ agonist	[66]
	5HT_2A_ partial agonist	[66]
	5HT_3A_ antagonist	[67]
	PPARγ agonist	[68]
	TRPA1 agonist	[56]
	TRPV1 agonist	[55,56]
	TRPV2 agonist	[50]
	TRPV3 agonist	[51]
	TRPM8 antagonist	[56]
	GABA_A_ positive allosteric modulator	[69]
	µ and δ opioid allosteric modulator	[52,53]
	GlyRα_1_ and α_3_ positive allosteric modulator	[70,71]
	AEA uptake inhibition by targeting FABPs	[55,56,57]
	Adenosine reuptake inhibitor	[58]
**CANNABIGEROL**		
**Chemical structure**	**Molecular Targets**	**References**
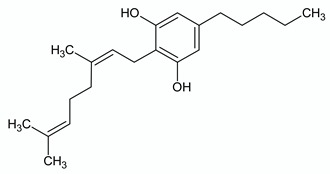	CB1 weak partial agonist	[72]
	CB2 weak partial agonist	[72,73]
	GPR55 antagonist	[74]
	α_2_-adrenoceptor agonist	[75]
	5HT1_A_ antagonist	[75]
	TRPA1 agonist	[49,56]
	TRPV1 agonist	[56]
	TRPV2 agonist	[56]
	TRPV4 antagonist	[51]
	TRPM8 antagonist	[49,56]
	Voltage-gated sodium channels Na_v_ blocker	[76]
	AEA uptake inhibitor	[56]
	MAGL inhibitor	[56]
**CANNABICHROMENE**		
**Chemical structure**	**Molecular Targets**	**References**
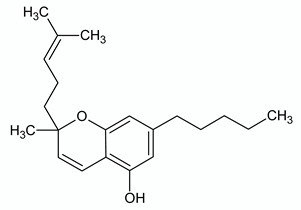	CB2 agonist	[77]
	TRPA1 agonist	[49,56]
	TRPV1 agonist	[56]
	TRPV3 agonist	[51]
	TRPV4 agonist	[51]
	TRPM8 weak antagonist	[56]
	AEA uptake inhibitor	[56]
	MAGL inhibitor	[56]
**CANNABIDIVARIN**		
**Chemical structure**	**Molecular Targets**	**References**
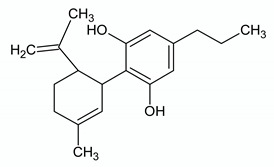	GPR55 antagonist	[74]
	TRPA1 agonist	[56]
	TRPV1 agonist	[56]
	TRPV2 agonist	[56]
	TRPV3 agonist	[51]
	TRPV4 agonist	[51]
	TRPM8 antagonist	[56]
	DAGL inhibitor	[56]
	AEA uptake inhibitor	[56]
**CANNABIDIOLIC ACID**		
**Chemical structure**	**Molecular Targets**	**References**
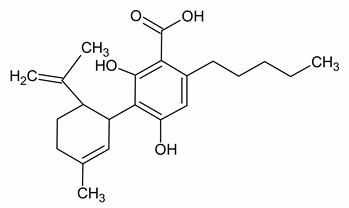	GPR55 antagonist	[74]
	5HT_1A_ agonist (potentiation)	[78]
	TRPA1 agonist	[49,56]
	TRPV1 weak agonist	[56]
	TRPM8 antagonist	[56]
	NAAA inhibitor	[56]
	DAGL inhibitor	[56]
	AEA uptake inhibitor	[56]
**CANNABIGEROLIC ACID**		
**Chemical structure**	**Molecular Targets**	**References**
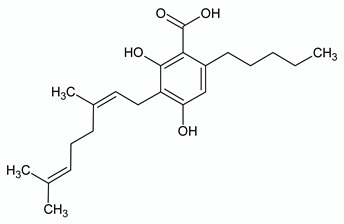	GPR55 inhibition	[74]
	TRPA1 weak agonist	[56]
	TRPV3 antagonist	[51]
	TRPV4 antagonist	[51]
	TRPM8 antagonist	[56]
	AEA uptake inhibitor	[56]
	DAGL inhibitor	[56]

See abbreviations at the end of the chapter.

**Table 3:** Main locations and functions of different components of the endocannabinoid system (ECS) within the gastrointestinal (GI) tract.

RECEPTORS		
COMPONENT	LOCATION	FUNCTION
**CB1** (binds AEA, 2-AG)	ENS: cholinergic neurons Mucosa: epithelial and plasma cells Vascular smooth muscle cells Lamina propria: macrophages and plasma cells	Reduces GI motility and secretion Modulates immune function
**CB2** (binds AEA, 2-AG)	ENS (under inflammatory conditions) Mucosa: epithelial cells, macrophages > plasma cells Lamina propria: macrophages and plasma cells	Reduces GI motility and secretion Modulates immune function
**TRPV1** (binds AEA > OEA)	Extrinsic afferent fibers, running through the muscle layers Immune cells adjacent to blood vessels.	Visceral hypersensitivity signaling Increase in intestinal contractility (under inflammatory conditions)
**PPAR-α** (binds AEA, 2-AG, OEA, PEA, others)	Enterocytes of the small intestine ENS Vagal afferent fibers Enteric glial cells	
**GPR55** (binds PEA)	Epithelial cells and ENS of the small intestine	
**GPR119** (binds OEA, PEA > AEA)	Villi: enteroendocrine L cells	Regulates the release of GLP-1
**METABOLIC ENZYMES**		
**COMPONENT**	**LOCATION**	**FUNCTION**
**FAAH**	Cells of the myenteric plexus in stomach and intestine	Degrades AEA, PEA, OEA
**MAGL**	Nerve cells and fibers in the muscle layers and mucosa of duodenum, ileum and colon	Degrades 2-AG Activity decreases from proximal to distal locations
**LIGANDS**		
**COMPONENT**	**LOCATION**	**FUNCTION**
**AEA**	Higher levels in colon than in ileum	Increased levels in IBD, celiac disease, diverticulitis, CRC
**2-AG**	Higher levels in ileum than in colon	Increased levels in CRC

See abbreviations at the end of the chapter.

**Table 4:** Anti-inflammatory activity of non-psychoactive phytocannabinoids in animal models of intestinal inflammation.

Compound	Model	Species (Strain, Sex)	Type of Treatment	Main Effects on Inflammation-Related Parameters	Reference
CBD	LPS	Mouse (Swiss OF1, males)	Preventive	↓ Enteric glial activation ↓ Mast cell activation ↓ macrophages activation ↓ TNF-α ↓ cleaved caspase-3 (↓ apoptosis)	[163]
CBD	LPS	Rat (Sprague Dawley, N.S.)	Preventive	↓ Histopathological alterations ↓ TNF-α ↓ IL-6 Restoration of smooth muscle myoelectrical activity	[132]
CBD	LPS	Mouse (C57/BL, N.S.)	Preventive	↓ IL-6 Restoration of UGT	[132]
CBD	TNBS	Mouse (CD1, males)	Preventive	↓ Histopathological alterations (i.p. treatment) No effects on histopathological alterations (oral treatment) ↓ Histopathological alterations (intrarectal treatment)	[164]
CBD	TNBS	Rat (Wistar, males)	Preventive	No effect on indices of inflammation ↓ MPO activity	[165]
CBD + THC	TNBS	Rat (Wistar, males)	Preventive	↓ Indices of inflammation ↓ MPO activity	[165]
CBD	DNBS	Mouse (ICR, males)	Preventive	↓ Indices of inflammation ↓ Histopathological alterations ↓ iNOS expression Normalization of IL-1β and IL-10 levels	[166]
CBD	DNBS	Mouse (ICR, males)	Curative	No effects on indices of inflammation	[131]
CBD (enriched botanical extract)	DNBS	Mouse (ICR, males)	Curative	↓ Indices of inflammation ↓ MPO activity	[131]
CBD (enriched botanical extract)	Croton oil	Mouse (ICR, males)	Curative	↓ Hypermotility (UGT)	[131]
CBD	Croton oil	Mouse (ICR, males)	Curative	↓ Hypermotility (UGT)	[131]
CBD	Croton oil	Mouse (ICR, males)	Curative	Normalization of hypermotility (UGT)	[127]
CBD	Croton oil	Mouse (ICR, males)	Curative	↓ Hypermotility (UGT)	[130]
CBG	DNBS	Mouse (ICR, males)	Preventive	↓ Indices of colitis	[167]
CBG	DNBS	Mouse (ICR, males)	Curative	↓ Indices of colitis ↓ Histopathological alterations Restoration of epithelial barrier function Normalization of IL-1β, IL-10 and IFN-γ levels Normalization of MPO activity Normalization of SOD activity	[167]
CBC	Croton oil	Mouse (ICR, males)	Curative	Normalization of UGT	[126]
CBC	DNBS	Mouse (ICR, males)	Curative	↓ Indices of colitis ↓ Histopathological alterations Amelioration of epithelial barrier function ↓ MPO activity	[168]
CBC	DNBS	Mouse (ICR, males)	Preventive	↓ Indices of colitis	[168]
CBDV	DNBS	Mouse (CD1, males)	Preventive	↓ Indices of colitis ↓ MPO activity Amelioration of epithelial barrier function ↓ Histopathological alterations	[128]
CBDV	DNBS	Mouse (CD1, males)	Curative	↓ Indices of colitis ↓ MPO activity Amelioration of epithelial barrier function ↓ Histopathological alterations Normalization of IL-1β, IL-6, and MCP-1α expression ↓ Inflammation-associated dysbiosis	[128]
CBDV	DSS	Mouse (CD1, males)	Curative	↓ Indices of colitis ↓ MPO activity ↓ IL-1β levels	[128]

See abbreviations at the end of the chapter.

## Abbreviations

2-AG	2-arachidonoylglycerol
2-AGE	Noladin ether, 2-arachidonoylglycerylether
5-HT_1A_	5-hydroxytryptamine 1A receptor
5-HT_2A_	5-hydroxytryptamine 2A receptor
5HT_3A_	5-hydroxytryptamine 3A receptor
A	Adenosine receptor
ABHD6/12	α/β -hydrolase domain 6/12
AEA	Anandamide, arachidonylethanolamide
AIDS	Acquired immunodeficiency syndrome
CB1	Cannabinoid receptor 1
CB2	Cannabinoid receptor 2
CBC	Cannabichromene
CBCA	Cannabichromenic acid
CBD	Cannabidiol
CBD-BDS	Cannabidiol-botanical drug substance
CBDA	Cannabidiolic acid
CBDV	Cannabidivarin
CBG	Cannabigerol
CBGA	Cannabigerolic acid
CBN	Cannabinol
CBNA	Cannabinolic acid
CBNDA	Cannabinodiolic acid
CD	Crohn’s disease
CDAI	Crohn’s disease activity index
CHS	Cannabinoid hyperemesis syndrome
CNS	Central nervous system
COX	Cyclooxygenase
CRC	Colorectal cancer
DAG	Diacylglycerol
DAGL	Diacylglycerol lipase
DEA	Drug Enforcement Administration
DNBS	Dinitrobenzene sulphonic acid
DSS	Dextrane sulfate sodium
ECS	Endocannabinoid system, endogenous cannabinoid system
EFSA	European Food Safety Authority’s
EMT	Endocannabinoid membrane transporter
ENS	Enteric nervous system
EU	European Union
FAAH	Fatty acid amide hydrolase
FABP	Fatty acid binding protein
FDA	Food and Drug Administration
FUFOSE	Functional Food Science in Europe
GABA	γ -aminobutyric acid
GABA_A_	γ-aminobutyric acid type A receptor
GI	Gastrointestinal
GLP-1	Glucagon like peptide 1
GlyR	Glycine receptor
GPR	G protein-coupled receptor
GPX	Glutathione peroxidase
IBD	Inflammatory bowel disease
IBS	Irritable bowel syndrome
IBS-C	Irritable bowel syndrome with constipation
IBS-D	Irritable bowel syndrome with diarrhea
IBS-M	Irritable bowel syndrome with mixed bowel habits
IFN	Interferon
IL	Interleukine
i.p.	Intraperitoneal
iNOS	Inducible nitric oxide synthase
LES	Lower esophageal sphincter
LOX	Lipooxygenase
LPI	Lysophosphatidylinositol
LPS	Lipopolysaccharide
MAGL	Monoacylglycerol lipase
MAPK	Mitogen-activated protein kinase
MCP-1α	Monocyte chemoattractant protein-1α
MPO	Myeloperoxidase
NAAA	N-acylethanolamine acid amide hydrolase, N-acylethanolamine hydrolyzing acid amidase
NADA	*N*-arachidonoyldopamine
NAPE	N-acyl-phosphatidylethanolamine
NAPE-PLD	N-acyl-phosphatidylethanolamine-specific phospholipase D
NO	Nitric oxide
O-AEA	Virodhamine, O-arachidonoylethanolamine
OEA	Oleylethanolamide
PEA	Palmithoylethanolamide
PNS	Peripheral nervous system
PPARα	Peroxisome proliferator-activated receptor type-α
PPARγ	Peroxisome proliferator-activated receptor type-γ
ROS	Reactive oxygen species
SOD	Superoxide dismutase
THC	Δ^9^-tetrahydrocannabinol
THCA	Tetrahydrocannabinolic acid
TNBS	Trinitrobenzene sulfonic acid
TNF	Tumor necrosis factor
TRP	Transient receptor potential
TRPA1	Transient receptor potential cation channel subfamily ankyrin 1-type
TRPM8	Transient receptor potential cation channel subfamily M member 8
TRPV1	Transient receptor potential vanilloid type-1 channel
TRPV2	Transient receptor potential cation channel subfamily V member 2
TRPV3	Transient receptor potential cation channel subfamily V member 3
TRPV4	Transient receptor potential cation channel subfamily V member 4
UC	Ulcerative colitis
UGT	Upper gastrointestinal transit
USDA	United States Department of Agriculture
XIAP	X-linked inhibitor apoptosis
Δ^9^-THC-BDS	Δ^9^-tetrahydrocannabinol-botanical drug substance
